# A p-n Heterojunction Based Pd/PdO@ZnO Organic Frameworks for High-Sensitivity Room-Temperature Formaldehyde Gas Sensor

**DOI:** 10.3389/fchem.2021.742488

**Published:** 2021-09-20

**Authors:** Faheem Ullah Khan, Shahid Mehmood, Shiliang Liu, Wei Xu, Muhammad Naeem Shah, Xiaojin Zhao, Junxian Ma, Yatao Yang, Xiaofang Pan

**Affiliations:** College of Electronics and Information Engineering, Shenzhen University, Shenzhen, China

**Keywords:** MOF templates, p-n heterojunction, Pd/PdO@ZnO nanomaterials, formaldehyde sensor, room temperature, fast response/recovery

## Abstract

As formaldehyde is an extremely toxic volatile organic pollutant, a highly sensitive and selective gas sensor for low-concentration formaldehyde monitoring is of great importance. Herein, metal-organic framework (MOF) derived Pd/PdO@ZnO porous nanostructures were synthesized through hydrothermal method followed by calcination processes*.* Specifically, porous Pd/PdO@ZnO nanomaterials with large surfaces were synthesized using MOFs as sacrificial templates. During the calcination procedure, an optimized temperature of 500°C was used to form a stable structure. More importantly, intensive PdO@ZnO inside the material and composite interface provides lots of p-n heterojunction to efficiently manipulate room temperature sensing performance. As the height of the energy barrier at the junction of PdO@ZnO exponentially influences the sensor resistance, the Pd/PdO@ZnO nanomaterials exhibit high sensitivity (38.57% for 100 ppm) at room temperature for 1-ppm formaldehyde with satisfactory selectivity towards (ammonia, acetone, methanol, and IPA). Besides, due to the catalytic effect of Pd and PdO, the adsorption and desorption of the gas molecules are accelerated, and the response and recovery time is as small as 256 and 264 s, respectively. Therefore, this MOF-driven strategy can prepare metal oxide composites with high surface area, well-defined morphology, and satisfactory room-temperature formaldehyde gas sensing performance for indoor air quality control.

## Introduction

Considering the time people spend indoors (e.g., up to 80% of an individual’s life span) (Abdullah et al., 2019; Kelly and Fussell, 2019; Majd et al., 2019) but there exist indoor air pollution (IAP) is a challenging and vital problem for people’s health. The presence of many IAP-related pollutants such as volatile organic compounds (VOCs) in the construction materials, consumer products, household furnishings, paints, and their insufficient ventilation from buildings are considered dangerous components for human survival (Huang et al., 2016; Na et al., 2019). It is estimated that the indoor concentrations of these pollutants are 5–10 times higher than outdoor concentrations (Ligotski et al., 2019; Sarigiannis et al., 2019). Therefore, high indoor concentrations of VOCs cause building sickness syndromes, such as headaches, nausea, throat, nasal pain, breathing difficulties, cough, asthma, skin irritation, vomiting, and eczema (Na et al., 2019; Nag, 2019). Similarly, exposure to various VOCs, including formaldehyde (HCHO), for prolonged time leads to leukemia and nasopharyngeal cancer due to its mutagenic nature (Hopkinson and Schofield, 2018; Möhner et al., 2019). In light of such circumstances, the Occupational Safety and Health Administration (OSHA) has estimated the limit for HCHO exposure is 0.75 ppm (Na et al., 2019). The precise assessment and rapid identification of concentrations of HCHO offers valuable information that can be further used to control the IAP. In addition, various conventional techniques such as gas chromatography (GC) (Zhu et al., 2019), high-performance liquid chromatography (HPLC) (Schenk et al., 2019), spectrophotometry (Möhlmann, 1985), polarography (Septon and Ku, 1982), and ion chromatography (Lorrain et al., 1981) have been used to measure gaseous HCHO. However, the conventional techniques required complex instrumentations, long sampling time, high expensive and large energy consumption are main factors limiting their usage in real-time monitoring (Chung et al., 2013; Vikrant et al., 2018a; Vikrant et al., 2018b). Therefore, the presence of large number of challenges attract the scientific society to develop novel chemiresistive based gas sensors with high sensitivity and selectivity (Moon et al., 2014; Vikrant et al., 2018a; Vikrant et al., 2018b).

Moreover, the development of various fast and portable gas sensors were based on varieties of material, such as nanostructures of a metal oxide semiconductor (MOS), especially zinc oxide (ZnO), due to low cost, easy synthesis strategies, and good sensing performance (Wang et al., 2003; Jing and Zhan, 2008; Yang et al., 2011). Therefore, different methods were employed to synthesize diverse morphology of ZnO nanomaterials, such as nanowires (Mascini et al., 2018), nanoflowers (Li et al., 2015), and nanorods (Aljaafari et al., 2020). On the other side, a high operating temperature is required to activate gas sensing sites (Huang et al., 2011; Wang et al., 2014), eliminating the possibility of being integrated with IC circuits or other flexible substrates. In addition, the introduction of noble metal maintaining the catalytic characteristics with electronic sensitization, where the MOS materials decorated noble metals have been used to improve the gas sensitivity at room temperature (Majhi et al., 2015; Yu et al., 2017; Sehgal et al., 2021). In this sequence, the integration of metal-MOSs materials have been a remarkable candidate for gas sensing in the last decade; however, they still suffer low sensing performance towards small-concentration (ppm level) VOCs (Li et al., 2015). Therefore, preparing efficient gas-sensitive nanomaterials *via* metal-organic frameworks (MOFs) is highly desirable for ppm-level at room-temperature detection. Wang et al. (2018) have reported the sensing of VOCs in ppm-level using the MOSs derived from MOF, where the introduction of metal-MOSs played significantly in improving the sensing of VOCs at ppm-level. In addition, the high porosity, tunable structure, and high active surface area maintaining functional organic ligands can be employed to enhance the interaction of gas molecules with the surface of the materials of the noble-metal@MOSs (Li et al., 1999; Phan et al., 2010; Zhai et al., 2014a).

This study adopted a facile and mechanistic approach to develop Pd@MOF-5 to form Pd/PdO@ZnO using the thermal decomposing process. The hydrothermal synthesis method was used to produce the porous ZnO from MOF-5 integrating Pd nanoparticles (NPs). The resultant Pd/PdO@ZnO nanomaterials represent greatly improved gas-sensing performance towards ppm-level of formaldehyde gaseous contents. Moreover, the selectivity of Pd/PdO/ZnO nanomaterials was satisfactory with evident results. Finally, the detailed mechanism contributing to the highly promoted efficiency of gas sensing is explained in detail.

## Experimental Section

### Materials and Methods

1,4-benzene dicarboxylic acid, zinc nitrate hexahydrate 99.99%, polyvinylpyrrolidone K30 98%, *N, N*-dimethylformamide 99.8% [HCON (CH_3_)_2_], ethyl alcohol 99.5% (CH_3_CH_2_OH) were purchased from Shanghai Aladdin Biochemical Technology Company Ltd. While Palladium (II) chloride (PdCl_2_) 99% was purchased from Sigma Aldrich. The Millipore-Q water (15 MΩ cm) was used as solvents in all the conducted experiments. The chemicals mentioned earlier were used in experiments without further purification.

### Synthesis of Pd-NPs

According to previous literature (Zhai et al., 2014a), Pd-NPs were synthesized with a slight modification. The Pd-NPs were obtained using borohydride (NaBH_4_) as a reduction agent. Palladium dichloride (PdCl_2_, 1 mmol) and 6 ml of hydrochloric acid (HCl, 0.2 mol Lˉ^1^) were dispersed in 10 ml de-ionized water and then stirred for 24 h. After that, NaBH_4_ (0.25 mol L^−1^) was freshly prepared and then added drop-wise into the mixture and stirred until the solution’s color remained unchanged. After the completion of the reaction, the resultant mixture was centrifuged at 12,000 rpm for 12 min. Pd-NPs were collected, washed several times with de-ionized water, and dried.

### Synthesis of Pd/PdO@ZnO Nanomaterials

According to the previous study (He et al., 2013), the Pd@MOF-5 precursors were synthesized with minor modification. Typically, 0.5 g of PVP was dissolved in 60 ml of ethanol/DMF mixture solution (5/3 in volume ratio). Then, in turn, 0.1088 g Zn(NO_3_)_2_.6H_2_O and 0.225 g H_2_DBC have dissolved in the PVP mentioned above solution. After thoroughly mixing the solution, a 2.25 ml (1 mM, PdCl_2_) solution was added to form a transparent solution under continuous stirring. The resulting uniform solution was transferred to an autoclave of 100 ml Teflon and put in the oven for 8 h at a constant temperature of 160°C. The collected powders were separated by centrifugation after cooled to room temperature, washed several times with DMF, and dried overnight at 75°C in a vacuum. The as-synthesized Pd@MOF-5 was then thermally decomposed at 500°C for 1 hour under air at a heating rate of 2°C min^−1^.

### Material Characterization

Characterization and X-ray diffraction (XRD) patterns of the prepared samples were conducted with a Rigaku-Miniflex-600 X-ray diffractometer to check the materials’ structure. High-resolution transmission electron microscopy (HR-TEM) of HITACHI (HT7700). Thermo Scientific™ ESCALAB™ Xi + X-ray Photoelectron Spectrometer (XPS) Microprobe was used for the chemical state analysis of the material. Energy-dispersive X-ray spectroscopy analysis was carried out using a dual-beam scanning electron microscope (FEI, model Scios 02). The electrochemical characterizations in electrochemical impedance spectroscopy (EIS) and Mott Schottky analysis were demonstrated via the Gamery Reference 3,000 (pine instrument United States) in the frequency range of 0.1 Hz–100 KHz. VOCs’ room-temperature measurement was performed in the Intelligent Gas Sensing Analysis System CGS-4TPs (Beijing Elite Tech. Co. Ltd.).

### Fabrication and Measurement of Gas Sensors

The measurements were conducted by an intelligent gas sensing analysis system (CGS 4-TPs, Beijing Elite Tech Co., Ltd., China). The system includes a data acquisition system, a 1.8 L vacuum chamber, and a set of probe adjustments. A sample of Pd/PdO@ZnO nanomaterials was dispersed in ethanol to form a paste. A sufficient amount of the paste was then dropped onto the Interdigitated Electrodes (IDs) specified working area. For proper adhesion to the coating material’s electrode surface, the fabricated electrodes were then dried overnight at a temperature of 160°C. The prepared sensor was connected with a current-voltage source and placed in a fixed position inside the chamber. To draw a stable response from the IDEs, a specifically designed connector was used. Initially, in the presence of air, the baseline resistance was recorded. The gas sensing measurement was carried out at room temperature. The sensor’s response was defined as (R_g_-R_a_) ∗100/R_a_, where Rg and Ra were electrical resistance of the Pd/PdO@ZnO nanomaterials in the presence of target gas and air, respectively (Liu et al., 2013). The response time and recovery time were defined as when sensor resistance changes 90% of Rg and Ra resistance during the target gas and air exposure.

## Results and Discussion

### Structural and Morphological Characteristics

**FIGURE 1:**
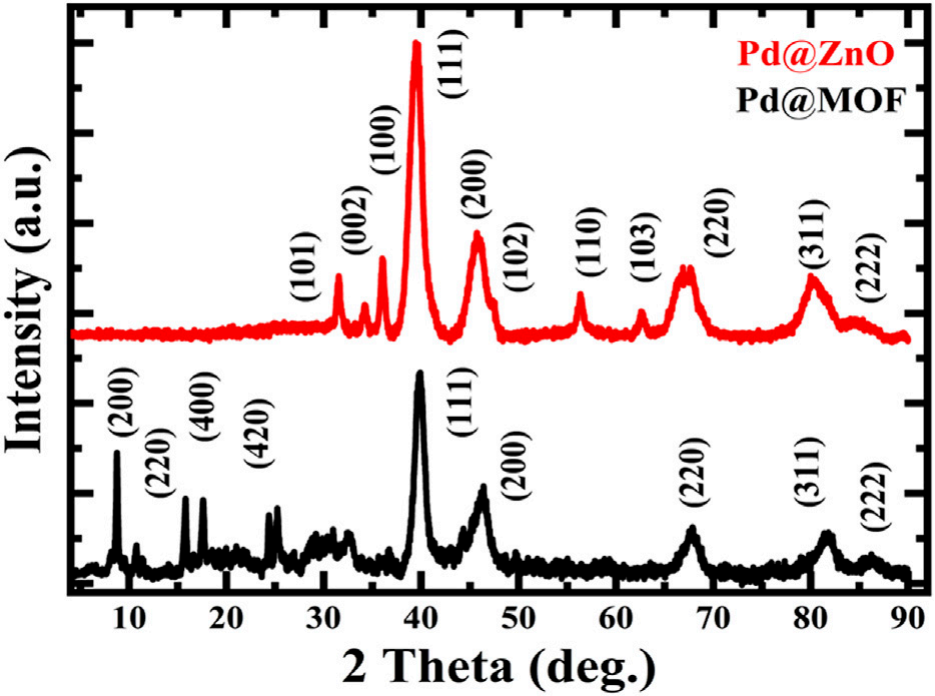
XRD patterns of Pd@ZnO and Pd@MOF nanomaterials.

Powder X-ray diffraction (XRD) analysis was used to study the crystal structure of Pd@MOF-5 nanostructure precursors and Pd@ZnO nanomaterials, as shown in Figure 1.

**GRAPHICAL ABSTRACT:**
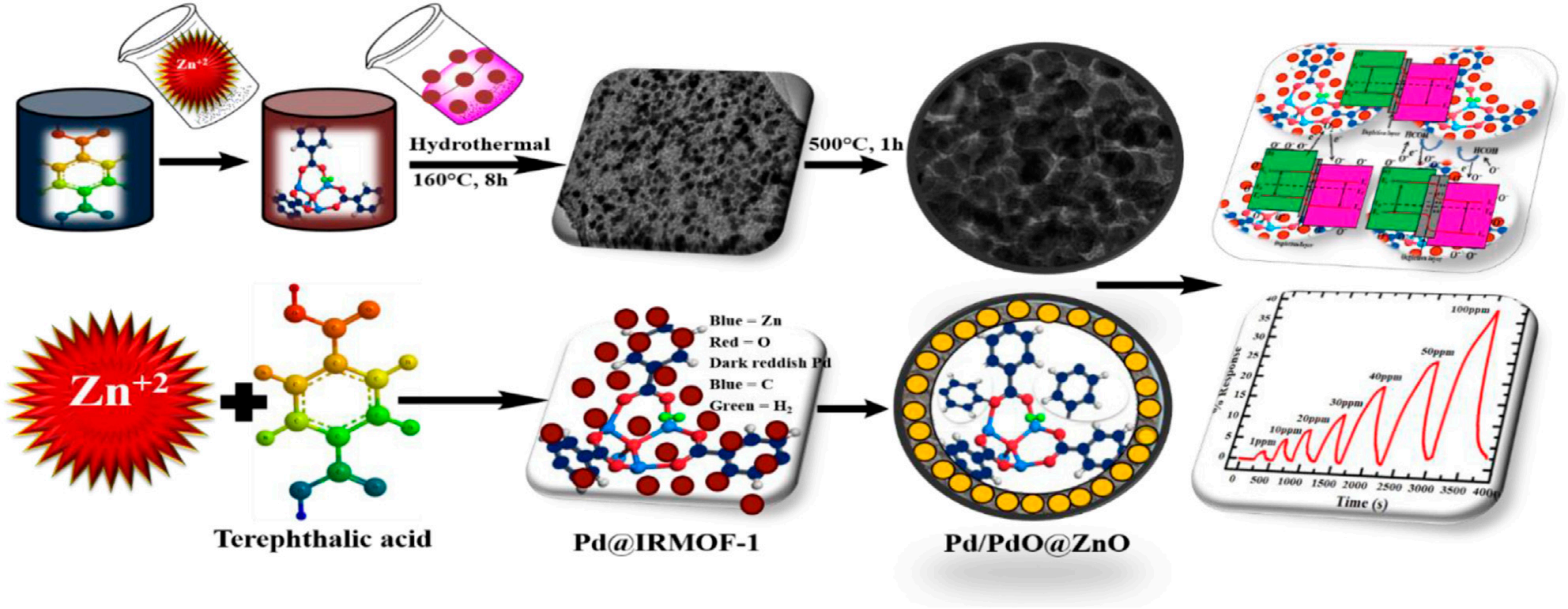


**FIGURE 2:**
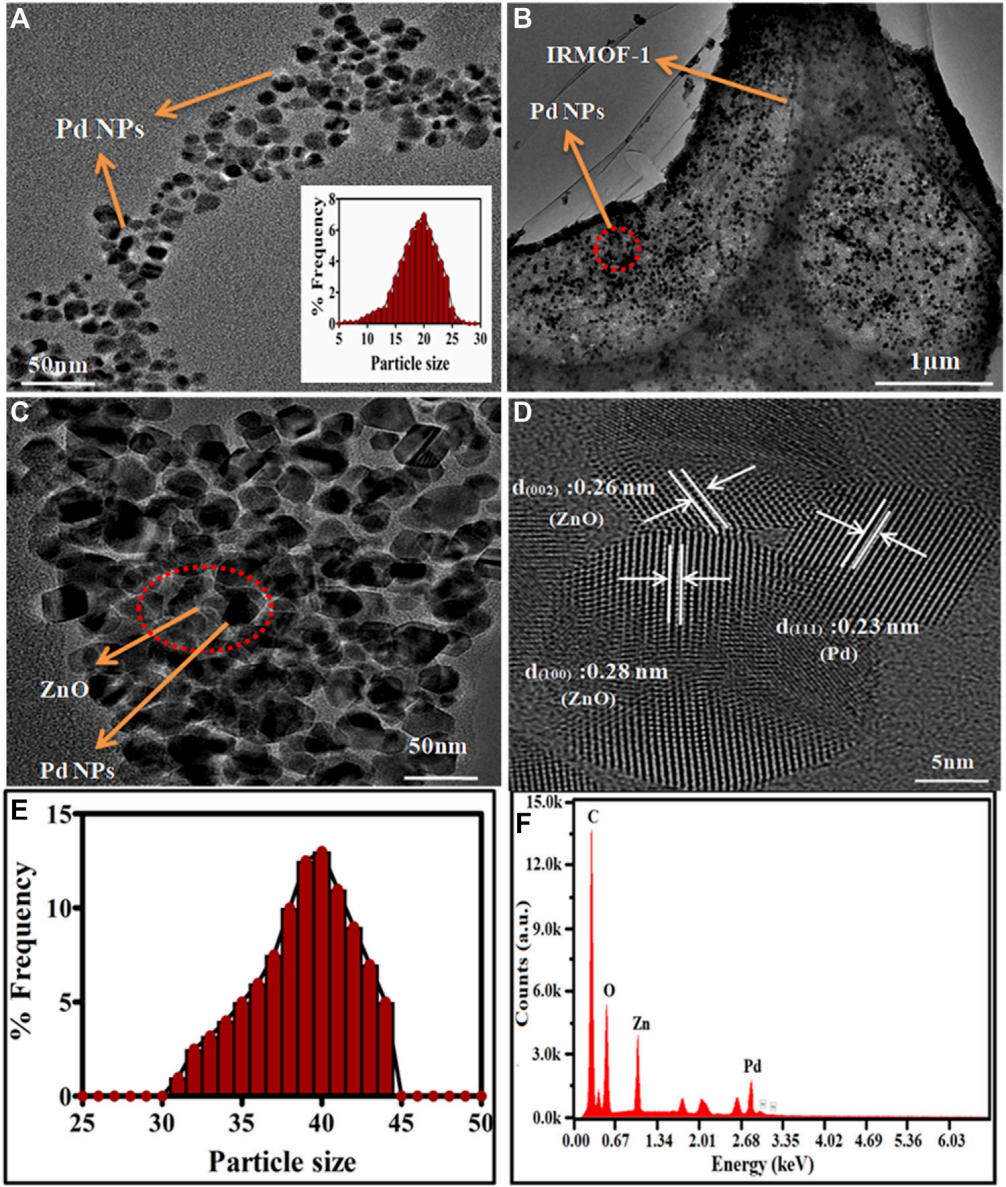
TEM images of **(A)** Pd-NPs (inset is about the size distribution), **(B)** Pd@MOF-5precursors, **(C)** Pd@ZnO nanomaterials, **(D)** HRTEM image of Pd@ZnO, **(E)** Size distribution of Pd@ZnO nanomaterials **(F)** EDX spectrum.

The Pd@MOF-5 precursor diffraction peaks were indexed to face-centered-cubic (fcc) Pd (JPCDS file no. 46–1,043) and crystalline MOF-5 in the previous study (Tranchemontagne et al., 2008). The MOF-5 shell changes to ZnO after pyrolysis with a hexagonal wurtzite structure (JCPDS file no. 36–1,451), and the Pd crystal structure was the same as the precursors. In addition, there is no indication of other dopants except diffraction peaks of Pd because of the high dispersion or excellent crystallinity property of Pd-NPs. Therefore, it could be confirmed that the nanocrystals are composed of fcc ZnO and fcc Pd. EDX is also used to investigate each component’s composition in Pd@ZnO nanoparticles, and the results are shown in Figure 2F. It could be observed from this figure that the materials were mainly composed of Zn and Pd elements.

The morphology of Pd-NPs and the Pd@ZnO nanomaterials were depicted in Figure 2. The TEM images of Pd-NPs in Figure 2A demonstrates most Pd-NPs have the symmetrical shape of a sphere-like structure, and the average particle size is 20 ± 5 nm. It can be seen from Figure 2B that the Pd@MOF-5 composite was aggregated together and has a smooth surface. Small Pd nanoparticles (about 20 nm) are highly dispersed on the inner surface of the MOF-5. The metal-organic frameworks immobilized the nanoparticles even after washing and sonication. Figure 2C represented the TEM micrograph of Pd@ZnO. The particle size distribution study from the TEM micrographs of Pd@ZnO was shown in Figure 2E, reveals an increase in average particle size with Pd loading from 20 to 40 nm, compared with Figure 2A. The HRTEM image in Figure 2D demonstrates the inter-plane distance of the Pd@ZnO nanoparticles lattice. The (111) planes of cubic Pd between two adjacent lattice places in the core are 0.23 nm. The lattice distances of 0.26 and 0.28 nm in the shell were assigned to (002) and (100) planes of the hexagonal wurtzite phase of ZnO.

The MOF-5 was formed as a secondary building unit by forming the bridge between Zn_4_(O) tetrahedron and H_2_BDC organic ligands. The MOF-5 structure collapses after pyrolysis, Zn ions turn into ZnO-NPs with the release of CO_2_ and H_2_O. The above findings demonstrated that Pd@ZnO nanomaterials maintain the nanostructures of the well-defined Pd@MOF-5 precursors. The EDX analysis of Pd@ZnO nanomaterials was also performed for a more detailed examination of atomic weight ratio, as shown in Figure 2F. The characteristic peaks of C, Pd, O, and Zn appear in Pd@ZnO nanomaterials, consistent with XRD and HRTEM characterization, indicating the successful synthesis of Pd@ZnO nanomaterials.

**FIGURE 3:**
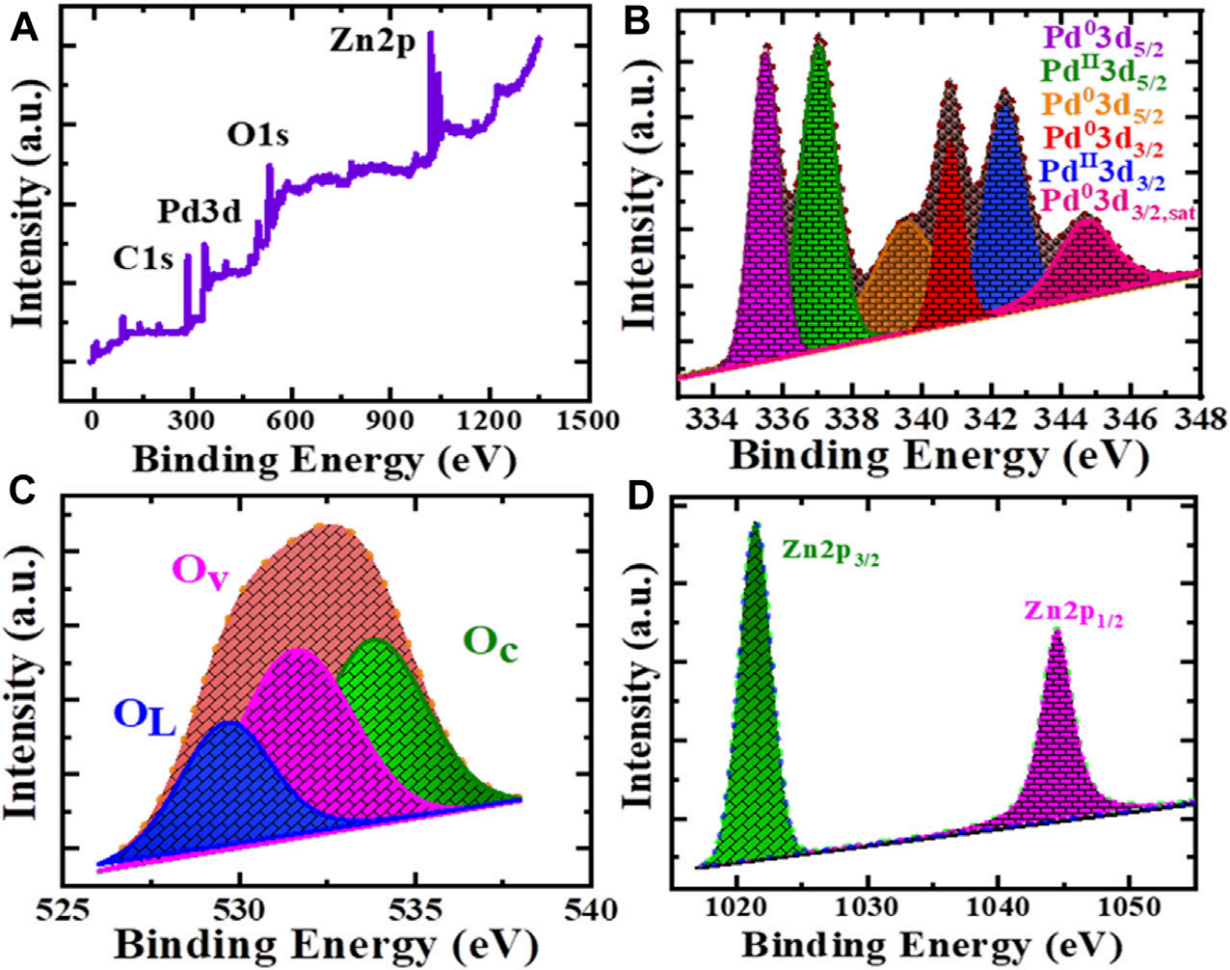
**(A)** XPS survey scan of Pd/PdO@ZnO nanomaterials. High resolution XPS of **(B)** Pd3d, **(C)** O1s and **(D)** Zn2p.

Using X-ray photoelectron spectroscopy (XPS), the chemical bonding states of Pd/PdO@ZnO nanomaterials were confirmed, as shown in Figure 3. Interestingly, comprehensive XPS survey scan spectra of Pd/PdO@ZnO nanomaterials in Figure 3A show firm peaks of Pd, Zn, and O species, which belong to the Pd/PdO@ZnO nanomaterials. The XPS spectrum of Pd_3_d in Figure 3B shows two pronounced bands at 335.4 and 339.3 eV, which can be assigned to 3d_5/2_ of metallic Pd and Pd/C, respectively (Xi et al., 2016). The other three peaks, 337, 340.4, and 342.4 eV, and one satellite peak at 344.4 eV, were attributed to the 3d_5/2_ and 3d_3/2_PdO and PdO/Pd, indicating that the surface of Pd-NPs is partially oxidized because of the exposure to the air. Therefore, the chemical state of palladium was Pd^0^ and Pd^2+,^ corresponding to Pd and PdO, respectively (Yamamoto et al., 1997; Zhong et al., 2019).

Furthermore, oxygen peaks in the O1s spectrum show three oxygen states located at 529.6, 531.6, and 533.8 eV, as shown in Figure 3C. The peak at 529.6 eV corresponds to lattice oxygen (O_L_), while peaks at 531.6 and 533.8 eV correspond to oxygen vacancies (O_v_) and absorbed oxygen species (O_c_), respectively. XPS of the O1s spectrum reveals that the adsorbed oxygen species and oxygen vacancies were essential for gas adsorption in gas sensing applications. The high-resolution spectrum of Zn2p in Pd/PdO@ZnO nanomaterials displays two typical peaks of 2p_1/2_ and 2p_3/2_ at binding energies 1,021.3 and 1,044.4 eV, corresponding to the binding energy of Zn^2+^ state Figure 3D (Chen et al., 2016). The Zn^2+^ state indicates that the Zn atoms were oxidized to ZnO in Pd/PdO@ZnO nanomaterials.

**FIGURE 4:**
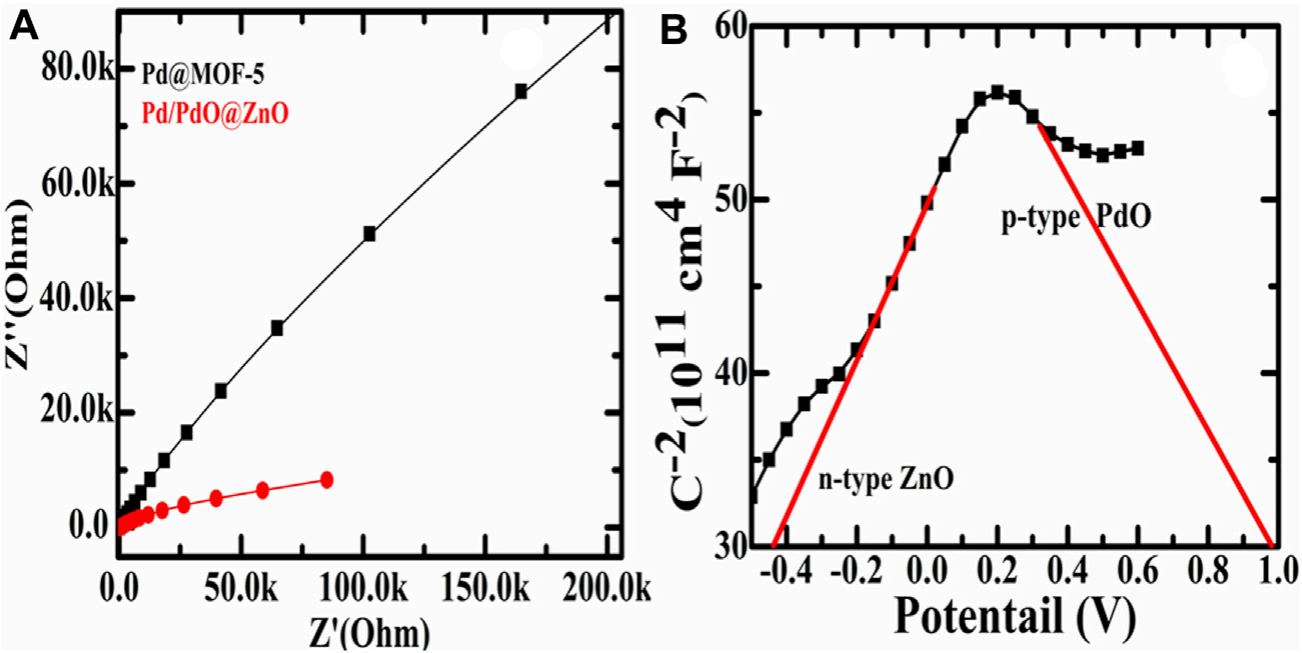
**(A)** Electrochemical impedance spectroscopy of Pd@MOF-5 and Pd/PdO@ZnO; **(B)** Mott-Schottky of n-type ZnO and p-type PdO.

Moreover, the as-prepared materials were electrochemically characterized through electrochemical impedance spectroscopy (EIS) to study the charge transport characteristics and the Mott Schottky effect. The EIS analysis of Pd@MOF-5 and Pd/PdO@ZnO predicts and determines the electron transfer kinetics in the presence of Na_2_SO_4_ as an electrolyte, as shown in Figure 4A. The EIS data was presented *via* the Nyquist plots of Pd@MOF-5 and Pd/PdO@ZnO, where there is a significant hindrance to the transport of charges in Pd@MOF-5 compared to the Pd/PdO@ZnO, and that determined the charge transfer resistance (R_ct_). A small R_ct_ indicates an improved charge transfer. Indeed, it can be found from Figure 4A that the R_ct_ values of the Pd/PdO@ZnO are less than Pd@MOF, indicating that the charge transfer resistance is lower, which indicates that the Pd/PdO@ZnO is conducive to rapid electron transfer, which leads to improved capacitance performance.

Furthermore, Figure 4B shows the Mott Schottky (MS) plot of Pd/PdO@ZnO to evaluate intrinsic properties such as flat band potential (V_fb_). Each point on the MS curve represents the value of capacitance at semiconductor electrolyte junction measured at respective potential (V) using the following Equation (Barma et al., 2020);

$${\text{C}}^{-2}=\left(\frac{2}{{\text{ε}}_{\text{s}}{\text{ε}}_{\text{o}}{\text{eN}}_{\text{D}}}\right)\left(\text{V}-{\text{V}}_{\text{fb}}-\frac{\text{kT}}{\text{e}}\right)$$

Where *ε*_o_ is the permittivity of free space, *ε*_s_ is the dielectric constant of the semiconductor, e is the electronic charge, k is Boltzmann’s constant, V is biased potential, V_fb_ is flat band potential, and T is the temperature (in Kelvin). Two regions can be observed in the MS curve of Pd/PdO@ZnO, one with the positive slope and the other with the negative slope. A positive slope indicates the n-type conductivity of ZnO and a negative slope with p-type conductivity of PdO. In addition, the V_fb_ for ZnO is −0.43 eV while for PdO is 0.96 eV. Emergences of two slopes in Pd/PdO@ZnO confirmed that the p-n heterojunction was formed between PdO and ZnO. Hence, the MS plots will assist in the determination of the charge carrier characteristics and their transport at the interface of electrode and electrolyte, as can be observed in Figure 4B. In addition, the applied voltage plays a key role in MS plots; the determination of charge characteristics will also assist in the formation of p-n heterojunction during injecting of gases *via* applying the voltage (Lasia, 2014).

### Gas Sensing Experiment

**FIGURE 5:**
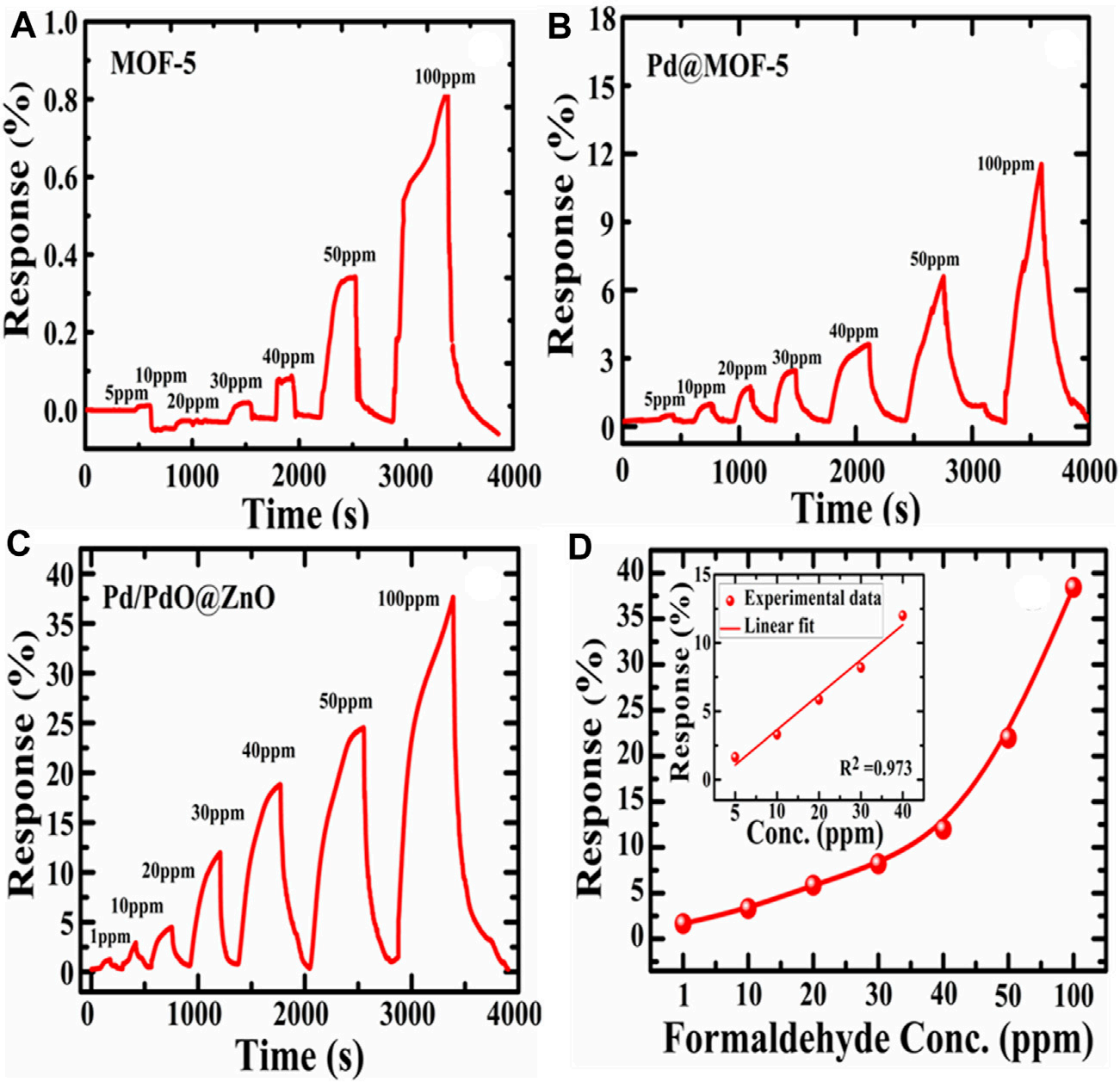
**(A–C)** Response and recovery curves of MOF-5, Pd@MOF-5, and Pd/PdO@ZnO towards formaldehyde, **(D)** response of Pd/PdO@ZnO toward various formaldehyde concentration inset shows the normalized response of Pd/PdO@ZnO as a function of the gas concentration of formaldehyde.

Sequential responses of MOF-5, Pd@MOF-5, and Pd/PdO@ZnO nanomaterials to 1, 10, 20, 30, 40, 50, and 100 ppm concentrations of formaldehyde were shown in Figures 5A–C. The response is defined as (R_g_-R_a_) ∗100/R_a_, where R_g_ is maximum resistance on target gas exposure while R_a_ is the minimum resistance in the air. However, it can be observed that the responses of the control MOF-5 and Pd@MOF-5 towards 5, 10, 20, 30, 40, 50, and 100 ppm concentrations of formaldehyde were lower than Pd/PdO@ZnO sensing nanomaterials to 1, 10, 20, 30, 40, 50, and 100 ppm concentrations of formaldehyde depicted in Figures 5A–C. The responses were 1.33, 2.66, 4, 5.71, 13.3, 20, and 38.57 for 1, 10, 20, 30, 40, 50, and 100 ppm formaldehyde, respectively. Pd/PdO@ZnO nanomaterials showed the most prominent response towards formaldehyde. It indicates that Pd/PdO@ZnO nanomaterials sensing materials have exhibited excellent sensing ability towards formaldehyde, as shown in Figure 5C. The response amplitude of sensing materials towards formaldehyde gases increases gradually with the increasing concentration of formaldehyde.

Figure 5D illustrates the relationships between the response and gas concentration. The increase towards formaldehyde in response is observed as the concentration increased to 100 ppm. Simultaneously, the detection limit of Pd/PdO@ZnO nanomaterials is 1 ppm. Figure 5D depicts a linear relationship between the sensitivity and concentration with formaldehyde less than 50 ppm. The sensitivity shows an exponential relationship on the further increase of the concentration, i.e., >50 ppm (Pawar et al., 2018). Pd/PdO@ZnO nanomaterials present themselves as promising materials for gas sensor applications.

The normalized response of the sensor with the gas concentration of formaldehyde is illustrated in the inset of Figure 5D. It can be concluded that the sensor response and the concentration of target gas are almost linearly related. The fitted equation for the sensor’s response as a function of concentration can be represented as response = m (concentration in ppm + c) where m is the slope, c is the intercept, and sum square residual (*R* ^2^), which is used to estimate the fitting quality. In the present case, the slope was 2.559, *R* ^2^ was 0.97. The normalized response of the sensor exhibits a linear fitting with formaldehyde concentration.

**FIGURE 6:**
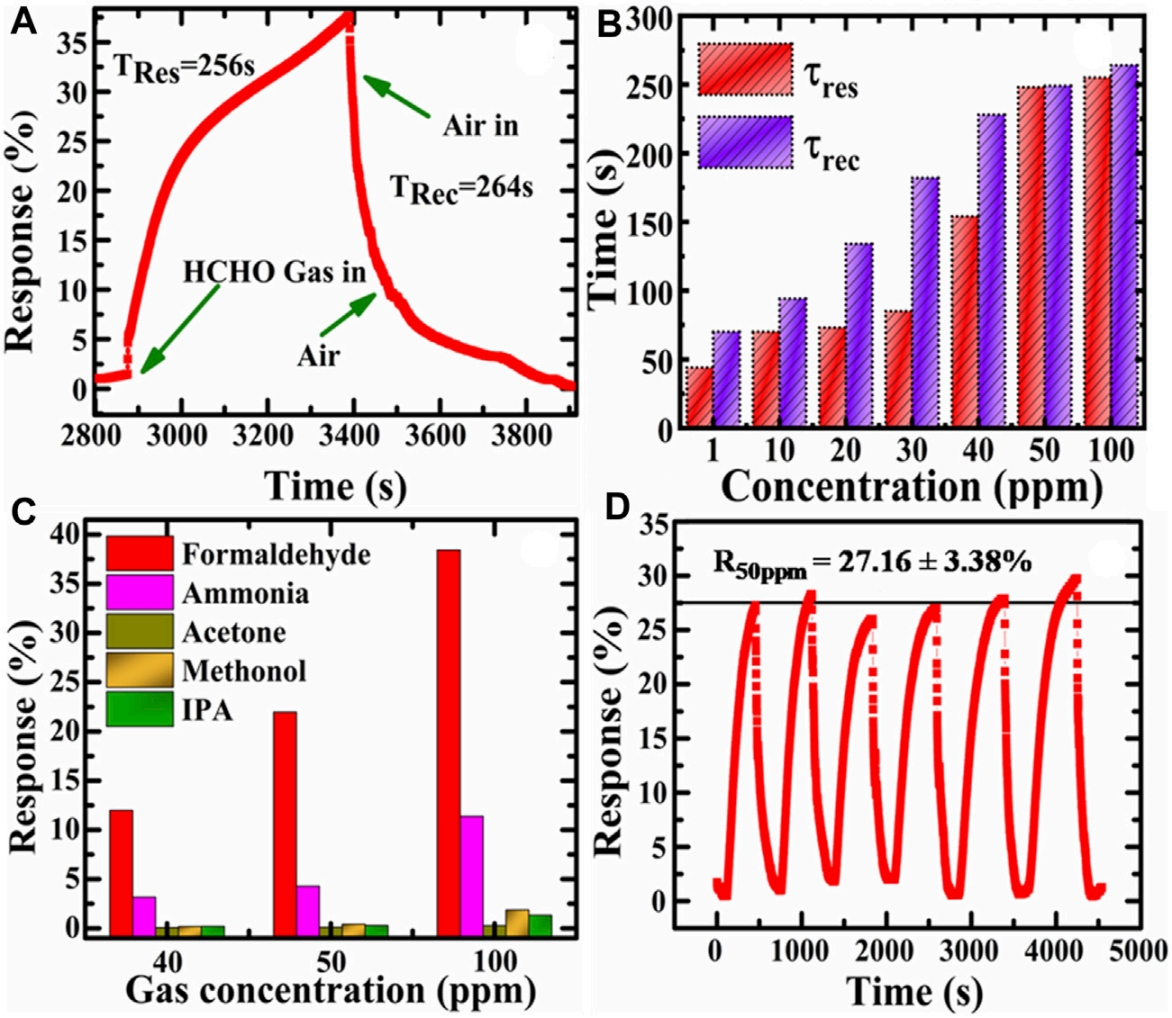
**(A)** Response and recovery time toward 100 ppm formaldehyde **(B)** response and recovery times for various formaldehyde concentrations, **(C)** the selectivity of Pd@ZnO for 1–100 ppm gaseous formaldehyde, ammonia, acetone, methanol, and IPA, and **(D)** Cyclic response studies of Pd@ZnO towards 50 ppm.

The response and recovery time is also an essential criterion of the gas sensors. The common practice for sensing speed is to describe the sensing response time (t_res_), which is the time required by the sensor to achieve 90% of the maximum resistance change after exposure to a given target gas concentration and the recovery time (t_rec_) as the time required for the sensor to recover 90% of the initial resistance when exposed to air. Figure 6A shows the dynamic response-recovery curves of the Pd/PdO@ZnO nanomaterials composite to 100 ppm HCHO at room temperatures. It can be seen that the resistance changes instantly when the sensor is exposed to HCHO. In this process, the time taken is only 256 s. The sensor is exposed to the air soon afterward to recover, and the time consumed by recovery is about 264 s. Figure 6B shows that the t_res_ increases from 44 to 256 s, and t_rec_ increased from 71 to 264 s for 1–100 ppm formaldehyde concentrations.

The selectivity analysis of the Pd@ZnO nanomaterials-based gas sensor was done by exposure to different kinds of common VOCs (Supplementary Figure S2). The results for five common VOCs opted as experimental comparison samples are shown in Figure 6C, indicating that gas sensors based on Pd/PdO@ZnO nanomaterials have negligible responses to other different kinds of VOCs. At the same time, there can be observed that much higher response to formaldehyde, respectively. Contrary to the gas sensors’ response to formaldehyde, it shows relatively low ammonia, acetone, methanol, and IPA responses. The higher sensing response to formaldehyde has been attributed to two reasons: the first is that VOCs’ volatilities and chemical properties are different. The second is that the different VOCs have different catalytic and adsorption performance towards Pd/PdO@ZnO nanomaterials-based gas-sensing materials. Formaldehyde response was 38.57%, while the response value for ammonia, acetone, methanol, and IPA were 11.47, 0.25, 1.83, and 1.19 for 100 ppm concentration, respectively.

In addition, it is possible to observe from Figure 6D that the important parameter of reproducibility was also investigated using the Pd/PdO@ZnO nanomaterials as sensing materials. It can be seen that Pd/PdO@ZnO sensing nanomaterials showed good reproducibility towards formaldehyde of 50 ppm at room temperature, where the response of the sensing materials after six cycles was 27.16 ± 3.38% display small deviation. We have also performed the reproducibility at 100 ppm of HCHO concentration (Supplementary Figure S1).

For comparison, we list the sensing results of Pd/PdO@ZnO nanomaterials and sensing materials reported elsewhere in Table 1. Looking at the results mentioned in Table 1, it can be concluded that the reported Pd/PdO@ZnO nanomaterials sensor has exhibited a higher response than the other reported sensors at room temperature. Although other groups have reported higher response formaldehyde but elevated temperature, the sensing response of 38.57% for 100 ppm was observed at room temperature in the present work. The presented room temperature sensing response is still higher than the response shown by the other group at room temperature.

**TABLE 1:** Comparison of the sensing performance of the Pd/PdO@ZnO sensor with previously reported formaldehyde sensors. Sensing mechanism of Pd/PdO@ZnO nanomaterials sensor.

S. No	Materials	Preparation method	Operating temperature (°C)	Concentration (ppm)	% Response	Ref.
1	rGO/MoS_2_	LbL	RT	15	3	Li et al. (2017)
2	Pt decorated	Hydrothermal	RT	200	39.3	Fu et al. (2019)
	MoO_3_nanowires					
3	PDA-GO	Hydrothermal, drop-casting	RT	300	24.3	Song et al. (2020)
4	rGO/SnO_2_	Hydrothermal	20	100	4	Zhang et al. (2017)
5	RGO/flower-like zinc	Hydrothermal, LBL, self-assembly	RT	45	10	Li et al. (2015)
6	1.5 at% Cr-doped	Co-precipitation method	200	50	82	Upadhyay et al. (2016)
	WO_3_nanosheet					
7	Fe-doped WO_3_ nanomaterials	Co-precipitation method	225	50	80	Upadhyay and Sahay, (2015)
8	CuO nanocubes	Thermal oxidation	300	3	—	Park et al. (2014)
9	NiO/NiFe_2_O_4_	Hydrothermal method	240	33.3–200	—	Xu et al. (2020)
10	Zn-doped NiO	Co-precipitation method	200	1.4	—	Fomekong et al. (2018)
11	Ag-LaFeO_3_	Sol-Gel method	90	1	25.2	Zhang et al. (2014a)
12	SnO/SnO2 nano-flowers	Hydrothermal method	120	50	80.9	Li et al. (2019)
13	Ag-LaFeO_3_	Molecular imprinting technique	40	0.5	—	Zhang et al. (2014b)
14	Pd/PdO@ZnO	Hydrothermal	RT	100	38.57	Present work

### Sensing Mechanism of Pd/PdO@ZnO Nanomaterials Sensor

The study of sensing materials detection towards the HCHO is quantitatively analyzed; therefore, when the sensor is exposed to air at room temperature, the oxygen molecules from the air will be adsorbed on the surface of active sensing material, forming ionized chemisorbed oxygen species (O^2-^, O^−^ or O_2_^−^). The introduction of Pd-NPs as a catalyst into the sensing material induce the crucial role in the formation of oxygen vacancies and further lead to the formation of depletion layer at the interface to sink the produced electrons and modulate the conductivity accordingly as well due to indispensable role of Pd-NPs in catalyzing surface sensing reactions (Yuasa et al., 2012; Zhai et al., 2014b). It has also been noticed in the XPS result that the oxidation of Pd to PdO increases the surface absorbed oxygen content in sensitive material (3d_5/2_ and 3d_3/2_ towards PdO and PdO/Pd, respectively in XPS spectra indicating that the surface of Pd-NPs is partially oxidized because of the exposure to the air), which are beneficial to improve the performance of sensors. More oxygen content can provide abundant active sites and lead to more direct adsorption of O_2_ molecules, thus raising the quantity of the adsorbed oxygen. Such XPS result indicates that incorporating PdO can increase the number of oxygen absorbed species, which is expected to contribute to the sensor response of the composite. Because of their high electronegativity, Chemisorbed oxygen species facilitates the capturing of free electrons from sensing material. When reducing gas, such as formaldehyde is introduced, possible reaction mechanisms at room temperature are shown in Eqs 1–3 to generate electrons.

$$O_{2}\left(\mathrm{gas}\right)\overset{\mathrm{yields}}{\to }O_{2}\left(\mathrm{ads}\right)$$ (1)

$$O_{2}\left(\mathrm{ads}\right)+e^{-}\overset{\mathrm{yields}}{\to }O_{2}^{-}\left(<100℃\right)$$ (2)

$$CH_{2}O+O_{2}^{-}\overset{\mathrm{yields}}{\to }CO_{2}+H_{2}O+2e^{-}\left(<100℃\right)$$ (3)

The possible chemical reaction described in Eq. 3 at room temperature is reversible and unstable. When air flows again, the above chemical reaction will be conducted in opposite reactions and ${\text{O}}_{2}^{-}$ are reproduced, and the latter surface attains its initial baseline resistance Ra.

**FIGURE 7:**
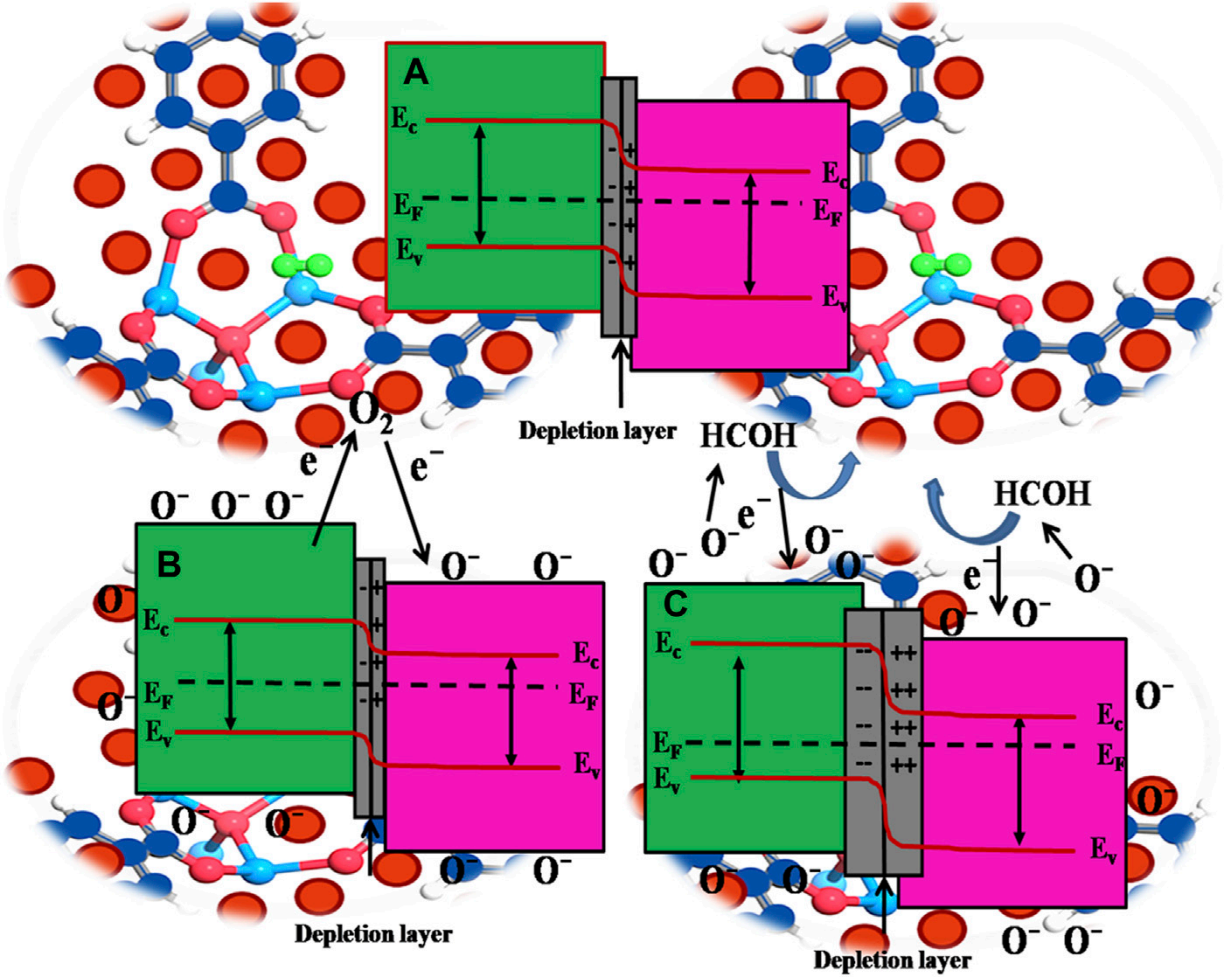
Bandgap diagram **(A)** Electronic depletion on the side of ZnO and depletion of the hole on the side of PdO **(B)** Smaller concentration of electrons decreases the recombination between the electrons and holes, leading to the thinned depletion region at the junction area and reduced resistance in the air **(C)** Larger number of holes need to flow from PdO to ZnO to neutralize the increased concentration of electrons at the depletion part of ZnO resulting in widening of the depletion region.

On the other side, the formation of p-n heterojunction also plays a crucial role in improving the sensing performance towards HCHO. In this regard, Mott Schottky provides us evidence that the p-type PdO (Majhi et al., 2019) and n-type of ZnO (Xia et al., 2019) lead to the formation of p-n heterojunction at the interface, which is possibly responsible for the enhancement of the gas sensing (Rakibuddin and Ananthakrishnan, 2015; Zhang et al., 2015; Park et al., 2016). When ZnO and PdO are brought in contact to form hetero-structure, electron transfer from ZnO to PdO to align the Fermi level (EF), leading to the formation of p-n heterojunction and charged free region (depletion layer) at the composite interface (electronic depletion on the side of ZnO and depletion of the hole on the side of PdO) as shown in Figure 7A.

The oxygen molecules in the air would like to adsorb on the material surface vacancy site when the nanomaterials are exposed to the air, and electrons are adsorbed during this reaction from oxide to form oxygen species. As a result, the concentration of carriers in ZnO is decreased. At the same time, that of the PdO side is increased (smaller concentration of electrons decreases the recombination between the electrons and holes), leading to the thinned depletion region at the junction area and reduced resistance in the air, as shown in Figure 7B. For formaldehyde, it can react with the oxygen species absorbed on the oxide surface. During the reaction, electrons can be released, indicating that more holes need to flow from PdO to ZnO to neutralize the increased concentration of electrons at the depletion part of ZnO. Therefore, the depletion layer of the PdO side hole is widened, and the resistance is increased, as shown in Figure 7C. More importantly, the depletion region’s width influences the energy barrier of the p-n heterojunction, which ultimately exponentially manipulates the resistance (Sun et al., 2019).

PdO then acts as a catalyst and prompts the dissociation of formaldehyde molecules into active radicals of H_2_O and CO_2_ through the spill-over effect, explained elsewhere in detail (Abdullah et al., 2015; Mhlongo et al., 2015; Shingange et al., 2016), while Pd plays a catalytic role activating the dissociation of molecular oxygen. During this process, to react with the adsorbed oxygen species, the formaldehyde molecules initially react with PdO before spilling over to the ZnO surface. As a result, additional oxygen species are thus created to react with the formaldehyde gas molecules and thus improve the sensing performance of formaldehyde after Pd loading.

Besides, the material structure and unique surface area are well known to affect the sensor’s sensing performance (Lee, 2009). The material’s porosity and large surface area enable more gas diffusion and abundant active adsorption and surface reaction sites (Wang et al., 2016).

## Conclusion

In this paper, metal-organic framework (MOF) derived Pd/PdO@ZnO porous nanostructures were synthesized by the hydrothermal method. TEM analysis confirmed that the size of the nanostructures was 40 nm. XPS studies revealed that metallic Pd (3d_5/2_), PdO (3d_5/2_), and PdO/Pd (3d_3/2_) chemical states of Pd were present. Besides, the formaldehyde gas sensing performance was thoroughly performed from 1 to 100 ppm at room temperature. The experimental results have revealed that Pd/PdO@ZnO novel nanomaterials exhibited a large response of 38.57% for 100 ppm formaldehyde at room temperature. The detection limit was 1 ppm with a fast response rate (256 s)/recovery rate (264 s) for 100 ppm. The main factors contributing to the enhanced gas-sensing performance were forming p-n heterojunction between PdO and ZnO interfaces and Pd catalytic property. Superior formaldehyde selectivity compared to other VOCs such as ammonia, acetone, methanol, and IPA can provide a new strategy to design new materials for formaldehyde detection.

## Data Availability

The original contributions presented in the study are included in the article/Supplementary Material, further inquiries can be directed to the corresponding authors.
