# Supplementary material for: A p-n Heterojunction Based Pd/PdO@ZnO Organic Frameworks for High-Sensitivity Room-Temperature Formaldehyde Gas Sensor
Source: Front Chem. 2021 Sep 20;9:742488. doi: 10.3389/fchem.2021.742488 (PMC8489732; doi:10.3389/fchem.2021.742488)
Supplement: Supplementary file 1 [file DataSheet1.PDF]

<u>Supplementary Data</u>

A p-n Heterojunction based Pd/PdO@ZnO Organic Frameworks for High-sensitivity Room-temperature Formaldehyde Gas Sensor

Faheem Ullah Khan, Shahid Mehmood, ShiliangLiu, Wei Xu, Muhammad Naeem Shah, Xiaojin Zhao, Junxian Ma, Yatao Yang*, Xiaofang Pan*

College of Electronics and Information Engineering, Shenzhen University, Guangdong Province, 518000, China.

***Correspondence:**(Xiaofang Pan, Yatao Yang)

email addresses: eexpan@szu.edu.cn, yatao86@163.com

Reproducibility test

It can be seen that the change in the six cycles was not significantly high that may not affect the selectivity of the designed sensor based on Pd/PdO@ZnO nanomaterials towards the HCOH of concentration(50 ppm)which displayed the reproducible results. The reproducibility cycles clearly show that the sensing represents the stable and reproducible results towards HCOH concentration of 50 ppm depicted in Fig. S1(a). We have also performed the reproducibility at 100 ppm of HCOH concentration, and the result is shown in Fig. S1(b).

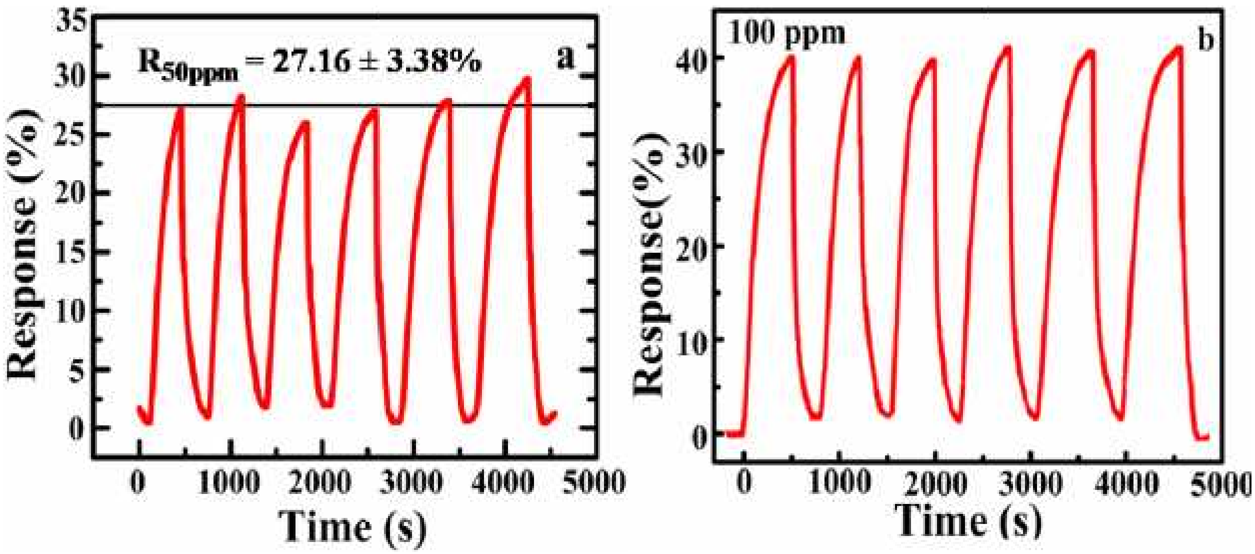

Fig. S1. The reproducibility of the sensing material Pd/PdO@ZnO under the optimized conditions towards Formaldehyde at 50 and 100 ppm gas concentrations (a-b).

Selectivity studies

The selectivity of sensing materials Pd/PdO@ZnO at long exposure (230 s) at 100 ppm of each gas concentration, where the selectivity result is added below shown in Fig. S2(a). However, there is no significant effect that can be observed in the result. Furthermore, we have considered your important comment and studied the selectivity analysis of higher and lower gas concentrations at 25,70 and 120 ppm for each interfering compound, as displayed in Fig.S2(b). There can be a slight difference observed in the response at higher and lower concentrations of gases rather than 100, 50, and 40 (ppm).

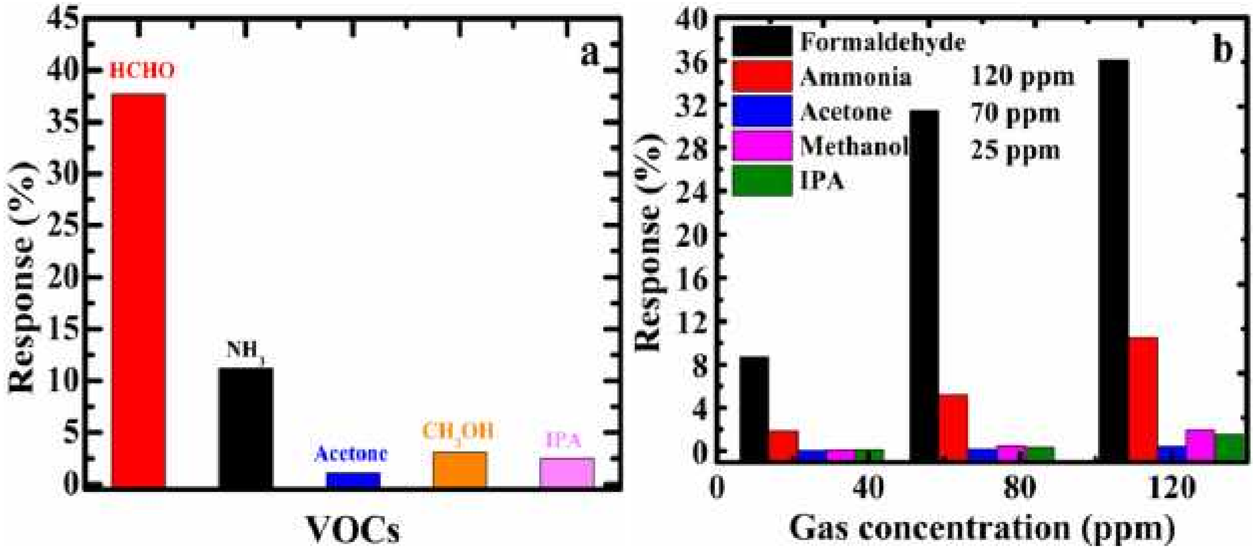

Fig. S2. (a) Selectivity of Pd/PdO@ZnO sensing material for 100 ppm gas concentration of various interfering gases at the long exposure time (230 s) (b); and Selectivity of Pd/PdO@ZnO sensing material for 25, 70, and 120 ppm gaseous of formaldehyde, ammonia, acetone, methanol, and IPA.
